# Spatial pattern and risk factors of resistance to important antibiotics among *E. coli* from veterans in seven U.S. Midwest states

**DOI:** 10.1017/ash.2025.10292

**Published:** 2026-01-28

**Authors:** Zhuo Tang, Qianyi Shi, Shinya Hasegawa, Margaret Carrel, Jacob Oleson, Michihiko Goto

**Affiliations:** 1 Department of Geosciences, Texas Tech University, Lubbock, TX, USA; 2 Department of Internal Medicine, University of Iowa, Iowa City, IA, USA; 3 School of Earth, Environment and Sustainability, University of Iowa, Iowa City, IA, USA; 4 Department of Biostatistics, University of Iowa, Iowa City, IA, USA

## Abstract

**Background:**

Effective antibiotic stewardship programing in clinical settings necessitates a good understanding of local prevalences of antimicrobial resistance and important patient and community risk factors. However, most studies are limited in sample size and geographic coverage.

**Methods:**

This study utilized phenotypic resistance data of *Escherichia coli* from the Veteran’s Health Administration of the United States (U.S.), incorporating 126,777 unique cultures from veteran outpatients from seven Midwest states from 2010 to 2023, to examine the spatial pattern and important individual- and county-level risk factors for resistance to four important classes of antibiotics. We utilized Bayesian conditional autoregressive zero-inflated Poisson regression models to generate smoothed rates of resistance in each county and multilevel logistic regression models to detect risk factors for resistance.

**Results:**

High overall rates of resistance were seen for fluoroquinolone (29%) and TMP-SMX (22%). Geographic variation was seen among and between antibiotic classes. Certain urban regions in the southern parts of Illinois, Indiana, and Ohio had higher local resistance rates for fluoroquinolone and TMP-SMX. Being male, having diabetes, and previous exposure to antibiotics are significant risk factors for all classes of antibiotics while the significance of other risk factors varied across classes.

**Conclusion:**

Diverse geographic patterns of resistance level may reflect differences in local prescribing practices, while the differential correlations with risk factors likely reflect their clinical indications and prescribing patterns in clinical settings. The local resistance rates and risk factors for different classes of antibiotics should provide important guidance in practicing empirical prescribing and antibiotic stewardship in clinical settings.

## Background

Appropriate antibiotic usage in outpatient settings is essential to preserve the utility of antibiotics.^1^ However, such optimized practice necessitates understanding local prevalence of antibiotic resistance and important patient and community risk factors associated with the likelihood of resistance. Currently, the understanding of the spatial patterns of antibiotic-resistant *E. coli* infections in the U.S. has been limited due to the lack of a unified nationwide surveillance system.^2^ Consequently, evidence from large regional or national studies that investigate the socioecological factors associated with antibiotic-resistant *E. coli* infections has been sparse and inconsistent. Among the existing literature, various factors including higher local temperature, precipitation, racial disparities, previous exposure to antibiotics, old age, and male sex, were identified to be positively associated with risks of resistance; however, the conclusions have been inconsistent.^3–12^ While the evidence indicates important potential contributing factors for resistance found in *E. coli* isolates in their respective regions, most studies had limited cases collected from a few local medical centers or a regional healthcare network rather than a nationwide healthcare system with substantial population coverage. In particular, antibiotic resistance research conducted the U.S. Midwest commonly focus on agricultural scenarios such as livestock feeding operations,^13^ ^,^ ^14^ highlighting the need for a more comprehensive understanding among non-agricultural communities. Thus, we proposed to answer the following questions: What are the spatial patterns of resistance against various classes of antibiotics among *E. coli* isolates obtained from veteran outpatients in the U.S. Midwest? And what individual- and community- level factors are associated with risks of having resistant-*E. coli* among veteran outpatients?

## Data and methods

To provide a stronger foundation for the evaluation of socioecological and climate drivers of drug-resistant *E. coli,* this study focused on seven Midwest U.S. states, a socio-demographically and environmentally heterogeneous region, and utilized phenotypic resistance data from the Veteran’s Health Administration (VHA), the largest integrated health care system in the U.S with broad geographic coverage. We selected the following classes of antibiotics as the focus of the study given their clinical importance in treating g bacterial infections: 3rd–5th generation parenterally administered cephalosporin (referred to as cephalosporin in the following text, unless the sub-class is particularly relevant); carbapenem; fluoroquinolone; sulfonamide; and trimethoprim.

**Figure 1.**
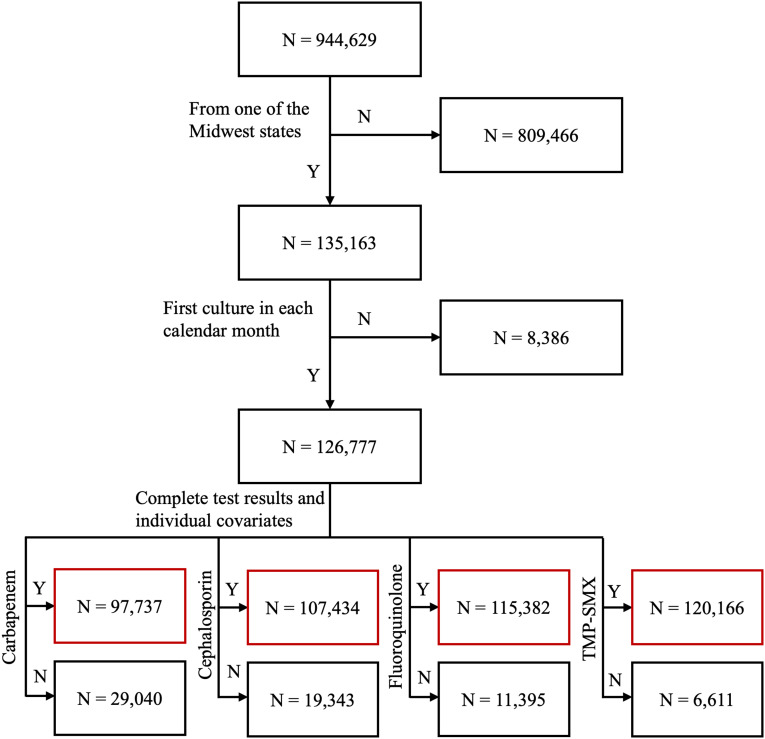
Flowchart of sample exclusion process. Number of samples included in the final models are labeled with red boxes.

This study utilized the data set from VHA Corporate Data Warehouse, examining 944,629 positive *E. coli* cultures collected in outpatient settings from those who received care in VHA outpatient clinics from January 1, 2010 to September 30, 2023. The data set incorporated the resistance testing results of more than 25 combinations of different types of antibiotics, among which the aforementioned five classes were analyzed. Among these classes, the test for sulfonamide and trimethoprim were combined as they are usually prescribed together (ie, TMP-SMX); we performed all subsequent analyses on 4 total classes. The data set also included the collection date of the sample, patients’ age, self-reported race/ethnicity, patient address, Elixhauser comorbidities, and previous exposure to different classes of antibiotics up to 90 days. Based upon the address information of patients from whom the cultures were taken, 135,163 were from one of the following Midwest states and were retained for analysis: Wisconsin, Minnesota, Iowa, Illinois, Indiana, Michigan, and Ohio. Additionally, we retained only the first culture in each calendar month for each patient to account for the bias introduced by duplicative samples, resulting in 126,777 unique samples. Among the samples, a small portion did not have complete test results or individual-level covariates. These samples were omitted in antibiotic class-specific models that corresponded with the missing data. The final sample sizes of the model of each class of antibiotics are as follows: n(carbapenem) = 97,737; n(cephalosporin) = 107,434; n(fluoroquinolone) = 115,382; n(TMP-SMX) = 120,166). Figure 1 shows the exclusion process from the original data set.

### Covariates selection

We included a range of individual- and county- level covariates in the analysis based upon their documented or potential association with resistant infections. For each individual, we included their age (categorized as below 45, 46–65, 66–85 and above 85), gender, race/ethnicity (as a binary category of non-Hispanic White vs other), comorbidity, and previous exposure to antibiotics (for the past 90 days). For the modeled comorbidities, we selected Chronic Obstructive Pulmonary Disease (COPD) and diabetes because of their long-term burden on one’s health as well as the fact that they are two of the most prevalent comorbidities among the veteran population. For county-level variables, we wished to account for the potential influence on resistance likelihood of both the natural and social environments. Thus, 5-year average temperature and precipitation from 2010 to 2014, retrieved from the NOAA National Centers for Environmental Information^15^ were included as two major environmental factors that impact bacterial population dynamics. We utilized the 5-year average as it was the longest time frame the data source provided. For social factors, we selected median household income to approximate the general living conditions and abundance of resources, as well as percent rural population to approximate local population density and occupation structures. The data for both variables were retrieved from 2010 U.S. Census data set via National Historical Geographic Information System.^16^ Table 1 shows all the variables included in the study.

**Table 1.** Variables and their respective data sources

Variables	Data resolution	Source
Resistant profile of *E. coli* samples	Individual level	VHA Corporate Data Warehouse
Race/Ethnicity		
Gender		
Age		
Comorbidity (COPD and Diabetes)		
Previous exposure to antibiotics		
5-year average temperature, 2010–2014	County level	NOAA National Centers for Environmental information, Climate at a Glance: County Mapping
5-year average precipitation, 2010–2014		
Median household income		2010 U.S. Census, via NHGIS
Rural/Urban status		

### Resistance risk mapping

To examine the spatial distribution of resistance against different classes of antibiotics, we mapped the risk of having a resistant culture in each county for each class of antibiotics. To account for the large number of zero counts of positive test results, we employed a zero-inflated Poisson regression model. To incorporate the spatial dependency among neighboring counties and reduce the instability introduced by sparse data in certain counties, we utilized a Leroux conditional autoregressive (CAR) prior to include a spatial random effect.^17,18^ The model was implemented using the CARBayes package^19^ in R version 2023.12.1.402^20^ to generate posterior predicted counts of positive resistance isolates in each county for the four classes of antibiotics. The predicted counts were then used to calculate the smoothed rates of resistance in each county, which were mapped using ArcGIS Pro (Esri, Redlands, CA). To allow for interpretation in clinically relevant settings but also fully exhibit the spatial variation of the rates, we utilized two different classification methods. First, we referred to a recent study where clinicians were surveyed about their perceived critical levels of resistance that may impact their prescription choices in different clinical settings.^21^ In accordance with study findings, we set 5%, 15%, 30%, and 50% as the critical thresholds of resistance. For the second method, we applied standard deviation classification (defined as the distance of the predicted value to the population mean measured by units of standard deviation of the population), which highlights counties whose resistance rates deviate substantially from the mean.

### Risk factor detection

To detect individual- and county-level factors that are associated with higher risks of resistant *E. coli* samples and simultaneously account for individuals being nested within counties, we employed a Bayesian multilevel logistic regression model using the *brms* package in R.^22^ Random intercepts were specified at county level to account for geographic heterogeneity, and default weakly informative priors were used for all parameters. It is worth noting that in each hierarchical model, the variable ‘recent exposure to antibiotics’ only included the class that is the same as the class used as the outcome. The convergence of the models was confirmed using & 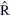 ;.

## Results

### Sample characteristics

Table 2 summarized resistance test results (n = 126,777) by gender, race/ethnicity, and age characteristics, comorbidity, and previous antibiotic exposure associated with each sample and the patient. It is worth noting that each patient may have more than one sample collected over the study period and thus be counted more than once in the summary. Table S1 summarized the number of unique patients in each antibiotic model since certain patients may be incorporated more than once. Around 78% of the samples were from a male patient, and 77% of them were from a non-Hispanic White patient. More than half of the samples were taken from a patient between the age of 66 and 85. Among the sample population, the highest rate of resistance was to fluoroquinolone (29.4%), with TMP-SMX resistance being the second highest (21.9%). Low levels of resistance were observed for cephalosporin (6.9%) and carbapenem which were particularly rare (0.3%). Ten percent of the samples were taken from patients with recent fluoroquinolone exposure; while 8% had TMP-SMX exposure, 2.6% had cephalosporin exposure and 0.8% had carbapenem exposure. 28.5% of the samples were from patients with COPD, and 38.9% from those with diabetes.

**Table 2.** The distribution of resistance for each class of antibiotics and by gender, race/ethnicity and age group, comorbidity and previous antibiotic exposure associated with each sample

Antibiotic Class	Carbapenem		Cephalosporin		Fluoroquinolone		Trimethoprim-Sulfamethoxazole	
	N n = 97,456	Y n = 275	N n = 100,029	Y n = 7,405	N n = 81,392	Y n = 33,990	N n = 93,787	Y n = 26,379
Gender								
Male	76,497 (99.7%)	250 (0.3%)	77,879 (92.4%)	6,444 (7.6%)	60,816 (67%)	29,369 (33%)	72,513 (77%)	21,269 (23%)
Female	20,959 (99.9%)	25 (0.1%)	22,150 (95.8%)	961 (4.2%)	20,576 (82%)	4,621 (18%)	21,274 (81%)	5,110 (19%)
Race/Ethnicity								
Non-hispanic white	74,392 (99.7%)	206 (0.3%)	76,568 (93.2%)	5,624 (6.8%)	63,912 (72%)	24,834 (28%)	73,245 (79%)	19,395 (21%)
Other	23,064 (99.7%)	69 (0.3%)	23,461 (92.9%)	1,781 (7.1%)	17,480 (66%)	9,156 (34%)	20,542 (75%)	6,984 (25%)
Age								
Below45	7,553 (99.8%)	12 (0.2%)	7,957 (96.1%)	327 (3.9%)	7,394 (84%)	1,414 (16%)	7,243 (79%)	1,954 (21%)
46–65	27,719 (99.7%)	80 (0.3%)	28,887 (94.5%)	1,673 (5.5%)	23,896 (73%)	8,895 (27%)	26,672 (78%)	7,405 (22%)
66–85	51,405 (99.7%)	151 (0.3%)	52,311 (92.2%)	4,442 (7.8%)	41,977 (69%)	19,226 (31%)	49,860 (78%)	13,980 (22%)
Above85	10,779 (99.7%)	32 (0.3%)	10,874 (91.9%)	963 (8.1%)	8,125 (65%)	4,455 (35%)	10,012 (77%)	3,040 (23%)
COPD								
Y	27,787 (99.7%)	93 (0.3%)	28,153 (91.6%)	2,588 (8.4%)	21,872 (66%)	11,111 (34%)	25,761 (75%)	8,558 (25%)
N	69,669 (99.7%)	182 (0.3%)	71,876 (93.7%)	4,817 (6.3%)	59,520 (72%)	22,879 (28%)	68,026 (79%)	17,821 (21%)
Diabetes								
Y	37,833 (99.6%)	135 (0.4%)	38,440 (91.8%)	3,418 (8.2%)	30,199 (67%)	14,704 (33%)	35,964 (77%)	10,890 (23%)
N	59,623 (99.8%)	140 (0.2%)	61,589 (93.9%)	3,987 (6.1%)	51,193 (73%)	19,286 (27%)	57,823 (79%)	15,489 (21%)
Antibiotic exposure (90 days)								
Y	857 (98.8%)	10 (1.2%)	2,278 (81%)	541 (19%)	4,080 (35%)	7,731 (65%)	5,676 (59%)	3,921 (41%)
N	96,599 (99.7%)	265 (0.3%)	97,751 (93.4%)	6,864 (6.6%)	77,312 (75%)	26,259 (25%)	88,111 (80%)	22,458 (20%)

### Risk mapping

**Figure 2.**
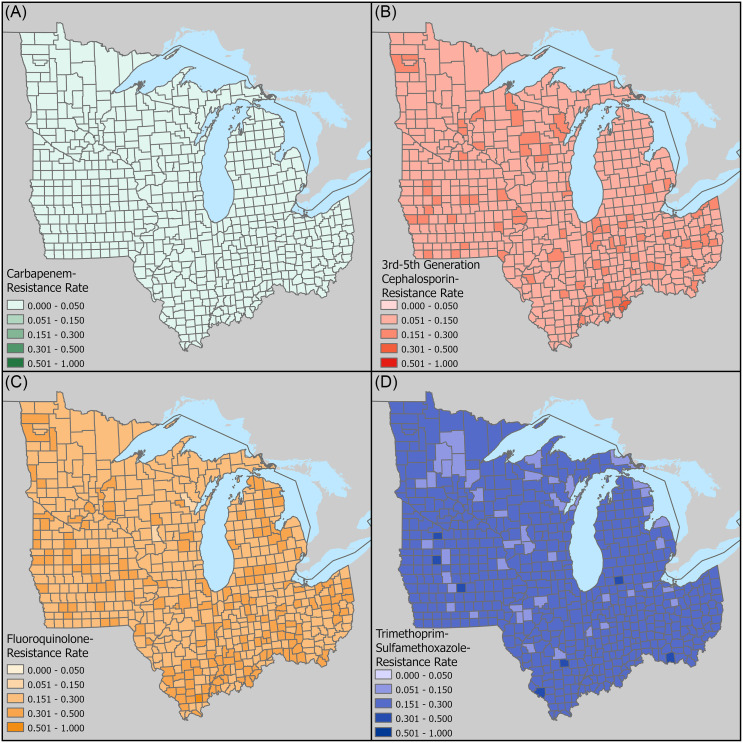
Map of spatially smoothed resistance risks among the seven Midwest states, using a unified classification of clinically critical thresholds according to Hasegawa, *et al.* ^21^ (A) carbapenem, (B) cephalosporin, (C) fluoroquinolone, (D) trimethoprim—sulfamethoxazole.

**Figure 3.**
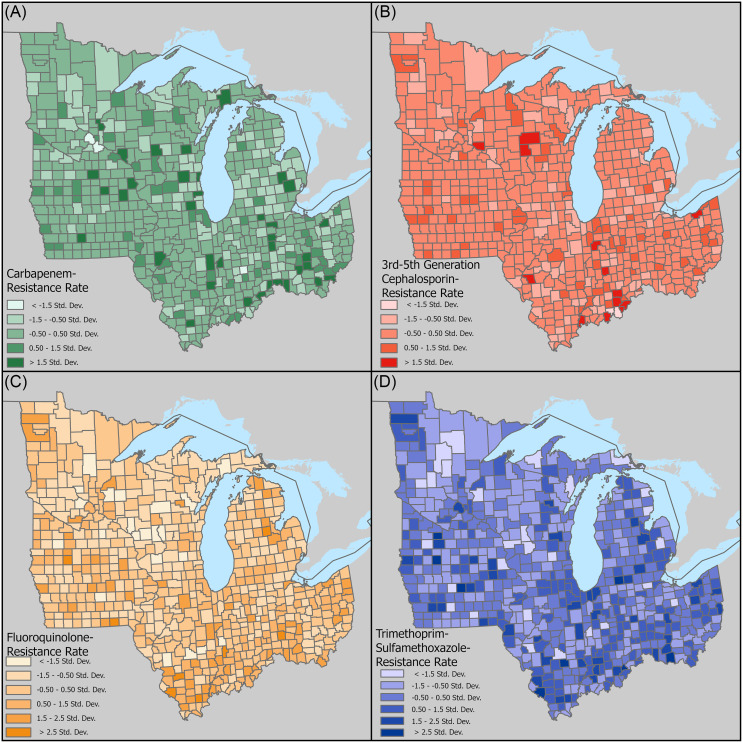
Standard deviation map of spatially smoothed resistance risks among the seven Midwest states. (A) carbapenem, (B) cephalosporin, (C) fluoroquinolone, (D) trimethoprim—sulfamethoxazole.

Figures 2 and 3 showed the spatial distribution using two different classification methods. The map using customized threshold intervals showed limited spatial variation, while the standard deviation map highlighted counties with resistance rates substantially higher or lower than the regional mean. Comparing the two maps, varying levels of resistance were seen across different classes of antibiotics as well as different counties.

Overall, carbapenem resistance rates were low with no counties exceeding 5%. However, certain urban areas including the cities of Milwaukee, Cincinnati, Columbus (Ohio), and suburban Detroit, had risks exceeding 1.5 standard deviations along with several rural counties such as Madison County, Iowa and St. Joseph County, Michigan.

For cephalosporins, most counties had resistance rates between 5% and 15%, with some areas reaching 15%–30%. Elevated risks clustered around urban areas including large metropolises including Clark and Floyd Counties of Indiana (adjacent to city of Louisville), Cuyahoga County, Ohio (city of Cleveland), and Marathon and Wood Counties, Wisconsin (city of Wausau), while a few rural counties also saw elevated risks.

Fluoroquinolone resistance was the most prevalent, ranging from 15% to 50%. Montgomery County, Ohio (city of Dayton) showed a particularly high rate. Other counties with high rates such as Saline, Clinton, and Randolph Counties in Illinois had around 50% rural populations. It also appeared that counties in the southern regions of the study area had generally higher risks of resistance.

Lastly, for TMP-SMX, most counties had resistance rates between 15% and 30%, with minor variations observed across the study area. Certain urban counties such as Story County, Iowa (city of Ames) had relatively high rates of resistance, while other high-risk counties appeared to be more rural.

### Risk factor detection

**Figure 4.**
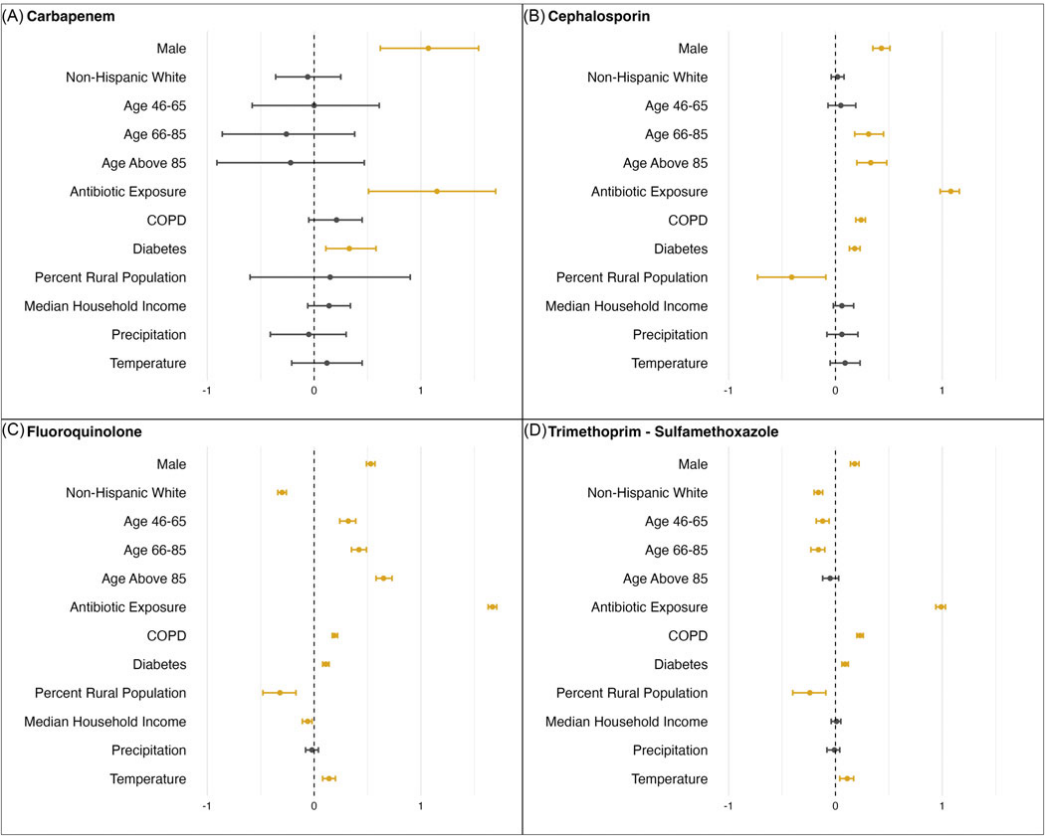
Posterior mean and 95% credible intervals of all covariates from Bayesian multilevel logistic regression models. Significant results were indicated using yellow color. The *x*-axes represent the coefficient generated from the model for each covariate.

Figure 4 and Table S2–5 exhibited the mean and 95% credible intervals of all covariates from the Bayesian multilevel logistic regression models across four classes of antibiotics. Table S2–5 included the exponentiated estimates (odds ratios) as well as & 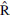 ; indicating model convergence. Statistical significance is determined using the 95% credible interval where an estimated interval not covering 0 is considered significant. Among the risk factors, being male, recent antibiotic exposure, and diabetes were consistent and significant risk factors across all four antibiotic classes. In addition, being above the age of 65 and having COPD were significant risk factors for developing cephalosporin-resistant *E. coli*, while having higher percentage of rural population in the county of residence was a significant protective factor. For fluoroquinolone-resistant *E. coli*, being above the age of 45, having COPD, and higher local average temperature were significant risk factors, while being non-Hispanic White, living in a more rural county and having higher median household income were protective factors. Finally, for TMP-SMX resistance, COPD and higher average temperature were additional risk factors. while being non-Hispanic White, age 46–85, and living in a more rural county.

## Discussion

Our study examined the spatial patterns and risk factors of resistant *E. coli* in outpatient settings among a Midwest veteran population against four important classes of antibiotics. Substantial spatial variations were seen between classes of antibiotics such the counties in the southern regions of the study area exhibiting elevated risks of fluoroquinolone resistance. Given the spatial pattern, practitioners are able take the residence of the patients into consideration when prescribing treatment. For example, clinicians may desire avoiding prescribing fluoroquinolone for outpatients in southern Midwest counties where the risk exceeds 30% depending on their risk tolerance, according to Hasegawa *et al.* ^21^ While we lack data to confirm the cause of these elevated risks, one of the reasons of the variations may be different levels of antibiotic consumption or particular resistant strains efficiently circulating among the local community. Such hypotheses could be further explored in combination with community antibiotic consumption data or phylogenetic analysis on the bacterial genomes.

Multi-level logistic regression models revealed important risk factors for resistant *E. coli* infections. Resistance was more common in males than females, consistent with prior large-scale studies.^10^ One potential reason is that urinary tract infections in males are more often complicated by anatomical differences and underlying urological abnormalities.^23^ Such complexity often requires prolonged or repeated treatment and the use of broad-spectrum antibiotics such as fluoroquinolone.^24^

Racial disparities in antibiotic resistance have been repeatedly documented for *E. coli* and other bacteria across diverse populations.^5–9^ In our study, non-Hispanic White was protective for fluoroquinolone and TMP-SMX resistance, but not for cephalosporin and carbapenem. Such difference could be due to that fluoroquinolone and TMP-SMX are commonly prescribed for outpatients,^25,26^ and the increased community exposure intersects with disparities experienced by minority veterans, including potentially more crowded living conditions, barriers to accessing VHA healthcare facility and higher health burdens including infectious diseases.^11,27^ In contrast, third- to fifth-generation parenterally administered cephalosporins and carbapenem are primarily used in institutionalized settings with standardized prescription criteria, which may diminish the racial disparity in their resistance.

Older age was significantly associated with higher risk of cephalosporin and fluoroquinolone resistance, consistent with prior evidence,^28^ potentially due to generally weaker immune system and repeated healthcare exposure. In contrast, older age appeared protective against TMP-SMX resistance. TMP-SMX has documented drug interactions with renin-angiotensin system inhibitors, which are commonly prescribed among older patients,^29,30^ and are of higher risks for patients with chronic kidney diseases,^31^ potentially influencing providers’ decisions and reducing exposure in this population.^32^

Recent exposure to the same class of antibiotics was the strongest predictor of resistance across all models. Such association has been widely documented in various studies and is not limited to *E. coli* or urinary tract infections.^12,33^ This highlights the substantial risk of prolonged or repeated exposure to antibiotics.

Finally, diabetes and COPD were risks factors for resistance across all four classes of antibiotics although the association between COPD and carbapenem resistance was not significant based on a 95% credible interval. It is well established that COPD and diabetes are associated higher risks of resistance through more frequent infections and subsequently more exposure to antibiotics as well as healthcare environments.^34,35^

Community-level covariates highlighted the impact of living environments. Counties with higher proportions of rural residents were negatively associated with cephalosporin, fluoroquinolone, and TMP-SMX resistance. Limited access to health care in rural settings may reduce the exposure to resistant bacterial strains, while higher population density in urban regions may facilitate pathogen transmission and more frequent prescription to antibiotics.^36^ Interestingly, its non-significant association with carbapenem may be due to the fact that carbapenems are mostly prescribed in hospital and much less frequently than the other agents, which may result in insignificant differences between urban and rural regions for other antibiotics;^37^ however, the effect of prescription in inpatient settings on community-acquired infections especially for reserved antibiotics needs further examination. In contrast, median household income is only negatively associated with fluoroquinolone resistance. While there seemed to be limited documentation explicitly about income, social deprivation has been associated with higher fluoroquinolone prescription rates in outpatient settings as a broad-spectrum agent, facilitating facilitates more empirical use without resistance testing.^38,39^ In socially deprived communities, the limited access to health care facilities and lower follow-up rates may lead to disproportional prescription decisions toward fluoroquinolone.^40^

Finally, higher local temperatures were associated with fluoroquinolone and TMP-SMX resistance. This aligns with previous findings^3^ that higher temperature may facilitate bacterial growth and horizontal gene transfer. This also reflects the higher rates of fluoroquinolone and TMP-SMX resistance observed in southern states. However, this association was not extended to cephalosporin or carbapenem. This again may be attributable to the fact that third- to fifth-generation parenterally administered cephalosporins and carbapenem are largely limited to institutionalized use and whose resistance levels are less sensitive to local environmental conditions. These risk factors provided important considerations for practitioners in clinical settings. Particularly, the community-level factors such as whether the patient reside in a rural or urban area help clinicians evaluate the risk of resistance, in addition to the more commonly considered factors such as previous antibiotic exposure.

Our study has several limitations. The veteran population is demographically skewed toward older, non-Hispanic White male with higher comorbidity burdens, limiting the generalizability of our results to non-veteran communities. In addition, infections treated empirically without culture confirmation as well as antibiotics administered outside of the VHA system were not captured in the data set, potentially underestimating certain cases and exposure, particularly for those deemed clinically less severe. Finally, our analysis was performed at county level, while community-level factors such as urban and rural statuses may be better captured at finer spatial scales. The observatory and ecological nature of our analysis also means that our results should be interpreted with caution and that causal connections hypothesized in our discussion needs to be confirmed with further examinations.

Nevertheless, our study represents one of the largest scales of similar studies in the existing literature to our knowledge. We identified geographic variation in antibiotic resistance, with certain counties exhibiting elevated risks. These patterns highlight the need for targeted antibiotic stewardship, particularly in areas with resistance rates have exceeding clinically significant thresholds. Our study also identified key risk factors for resistance across classes of antibiotics. Notably, the variation in these factors across antibiotic classes likely reflects differences in their clinical indications and prescribing patterns. For example, antibiotics that are prescribed more in outpatient scenarios such as fluoroquinolone and TMP-SMX are more sensitive to environmental and socioeconomic factors compared to those mostly used in institutionalized settings. The local resistance rates, as well as individual- and county-level risk factor for different classes of antibiotics, should provide important guidance in practicing antibiotic stewardship in clinical settings. Future work should expand study population and incorporate additional comorbidities and contextual risk factors.

## Supporting information

10.1017/ash.2025.10292.sm001 Tang et al. supplementary material Tang et al. supplementary material

## Data Availability

The datasets generated in this study are not publicly available due to their sensitive nature and ownership by the VHA.

## Supplementary material

The supplementary material for this article can be found at https://doi.org/10.1017/ash.2025.10292
