# Supplementary material for: Spatial pattern and risk factors of resistance to important antibiotics among E. coli from veterans in seven U.S. Midwest states
Source: Antimicrob Steward Healthc Epidemiol. 2026 Jan 28;6(1):e35. doi: 10.1017/ash.2025.10292 (PMC12854879; doi:10.1017/ash.2025.10292)
Supplement: Tang et al. supplementary material [file S2732494X25102921sup001.docx]

**Supplementary Material**

Table S1 Number of unique patients in each model constructed for different antibiotic class.

| Antibiotic Class | Number of Unique Patients | Number of Patients with More than One Record |
| --- | --- | --- |
| Carbapenem | 55855 | 18064 |
| Cephalosporin | 61037 | 19880 |
| Fluoroquinolone | 65241 | 21493 |
| Trimethoprim - Sulfamethoxazole | 67370 | 22400 |

Table S2 Posterior means, 95% credible intervals, corresponding odds ratios, and convergence diagnostics (R̂) for all model covariates in the Bayesian logistic regression model of carbapenem resistance. Significant results are labeled with an asterisk.

| **Variable** | **Posterior Mean (Odds Ratio)** | **95% Credible Interval (Odds Ratio)** | **R̂** |
| --- | --- | --- | --- |
| Male | 1.07 (2.92)* | 0.62, 1.54 (1.86, 4.66)* | 1.00 |
| Non-Hispanic White | -0.06 (0.94) | -0.36, 0.25 (0.70, 1.28) | 1.00 |
| Age 46-65 | 0.00 (1.00) | -0.58, 0.61 (0.56, 1.84) | 1.00 |
| Age 66-85 | -0.26 (0.77) | -0.86, 0.38 (0.42, 1.46) | 1.00 |
| Age Above 85 | -0.22 (0.80) | -0.91, 0.47 (0.40, 1.60) | 1.00 |
| Antibiotic Exposure | 1.07 (3.16)* | 0.51, 1.70 (1.67, 5.47)* | 1.00 |
| COPD | 0.21 (1.23) | -0.05, 0.45 (0.95, 1.57) | 1.00 |
| Diabetes | 0.33 (1.39)* | 0.11, 0.58 (1.16, 1.79)* | 1.00 |
| Percent Rural Population | 0.15 (1.16) | -0.60, 0.90 (0.55, 2.46) | 1.00 |
| Median Household Income | 0.14 (1.15) | -0.06, 0.34 (0.94, 1.40) | 1.00 |
| Precipitation | -0.05 (0.95) | -0.41, 0.30 (0.66, 1.35) | 1.00 |
| Temperature | 0.12 (1.13) | -0.21, 0.45 (0.81, 1.57) | 1.00 |

Table S3 Posterior means, 95% credible intervals, corresponding odds ratios, and convergence diagnostics (R̂) for all model covariates in the Bayesian logistic regression model of cephalosporin resistance. Significant results are labeled with an asterisk.

| **Variable** | **Posterior Mean (Odds Ratio)** | **95% Credible Interval (Odds Ratio)** | **R̂** |
| --- | --- | --- | --- |
| Male | 0.43 (1.54)* | 0.35, 0.51 (1.42, 1.67)* | 1.00 |
| Non-Hispanic White | 0.02 (1.02) | -0.04, 0.08 (0.96, 1.08) | 1.00 |
| Age 46-65 | 0.05 (1.05) | -0.07, 0.19 (0.93, 1.21) | 1.00 |
| Age 66-85 | 0.31 (1.36)* | 0.18, 0.45 (1.20, 1.57)* | 1.00 |
| Age Above 85 | 0.33 (1.53)* | 0.20, 0.48 (1.22, 1.62)* | 1.00 |
| Antibiotic Exposure | 1.08 (2.94)* | 0.98, 1.16 (2.66, 3.19)* | 1.00 |
| COPD | 0.24 (1.27)* | 0.19, 0.28 (1.21, 1.32)* | 1.00 |
| Diabetes | 0.18 (1.20)* | 0.13, 0.23 (1.14, 1.26)* | 1.00 |
| Percent Rural Population | -0.41 (0.66)* | -0.73, -0.09 (0.48, 0.91)* | 1.02 |
| Median Household Income | 0.06 (1.06) | -0.02, 0.17 (0.98, 1.19) | 1.00 |
| Precipitation | 0.06 (1.06) | -0.08, 0.21 (0.92, 1.23) | 1.00 |
| Temperature | 0.09 (1.09) | -0.05, 0.23 (0.95, 1.26) | 1.01 |

Table S4 Posterior means, 95% credible intervals, corresponding odds ratios, and convergence diagnostics (R̂) for all model covariates in the Bayesian logistic regression model of fluoroquinolone resistance. Significant results are labeled with an asterisk.

| **Variable** | **Posterior Mean (Odds Ratio)** | **95% Credible Interval (Odds Ratio)** | **R̂** |
| --- | --- | --- | --- |
| Male | 0.53 (1.70)* | 0.49, 0.57 (1.63, 1.77)* | 1.00 |
| Non-Hispanic White | -0.30 (0.74)* | -0.34, -0.26 (0.71, 0.77)* | 1.00 |
| Age 46-65 | 0.32 (1.38)* | 0.24, 0.39 (1.27, 1.48)* | 1.00 |
| Age 66-85 | 0.42 (1.52)* | 0.35, 0.49 (1.42, 1.63)* | 1.00 |
| Age Above 85 | 0.65 (1.92)* | 0.58, 0.73 (1.79, 2.08)* | 1.00 |
| Antibiotic Exposure | 1.67 (5.31)* | 1.63, 1.71 (5.10, 5.53)* | 1.00 |
| COPD | 0.19 (1.21)* | 0.17, 0.22 (1.19, 1.25)* | 1.00 |
| Diabetes | 0.11 (1.12)* | 0.08, 0.14 (1.08, 1.15)* | 1.00 |
| Percent Rural Population | -0.32 (0.73)* | -0.48, -0.17 (0.62, 0.84)* | 1.00 |
| Median Household Income | -0.06 (0.94)* | -0.11, -0.02 (0.90, 0.98)* | 1.00 |
| Precipitation | -0.02 (0.98) | -0.08,0.04 (0.92, 1.04) | 1.00 |
| Temperature | 0.14 (1.15)* | 0.08, 0.2 (1.08, 1.22)* | 1.00 |

Table S5 Posterior means, 95% credible intervals, corresponding odds ratios, and convergence diagnostics (R̂) for all model covariates in the Bayesian logistic regression model of TMP-SMX resistance. Significant results are labeled with an asterisk.

| **Variable** | **Posterior Mean (Odds Ratio)** | **95% Credible Interval**  **(Odds Ratio)** | **R̂** |
| --- | --- | --- | --- |
| Male | 0.18 (1.20)* | 0.14, 0.22 (1.15, 1.25)* | 1.00 |
| Non-Hispanic White | -0.16 (0.85)* | -0.20, -0.12 (0.82, 0.89)* | 1.00 |
| Age 46-65 | -0.12 (0.89)* | -0.18, -0.06 (0.84, 0.94)* | 1.00 |
| Age 66-85 | -0.16 (0.85)* | -0.23, -0.10 (0.80, 0.91)* | 1.00 |
| Age Above 85 | -0.05 (0.95) | -0.12, 0.03 (0.89, 1.03) | 1.00 |
| Antibiotic Exposure | 0.99 (2.69)* | 0.94, 1.03 (2.56, 2.80)* | 1.00 |
| COPD | 0.23 (1.26)* | 0.20, 0.26 (1.22, 1.30)* | 1.00 |
| Diabetes | 0.09 (1.09)* | 0.06, 0.12 (1.06, 1.13)* | 1.00 |
| Percent Rural Population | -0.24 (0.79)* | -0.40, -0.09 (0.67, 0.91)* | 1.00 |
| Median Household Income | 0.01 (1.01) | -0.04, 0.05 (0.96, 1.05) | 1.00 |
| Precipitation | -0.01 (0.99) | -0.08, 0.04 (0.92, 1.04) | 1.00 |
| Temperature | 0.11 (1.12)* | 0.04, 0.17 (1.04, 1.19)* | 1.00 |
